# Progress in Disease of Digestive Organs

**Published:** 1903-06-27

**Authors:** 


### Progress in Disease of Digestive Organs.

(*Concluded from page* 211.)

**Biliary Cirrhosis.**—P. Lereboulet [22] classifies the various types as follows:—(1) Hypertrophic or Hanot's type, where the liver and spleen are both enlarged, but the former is more affected than the latter; (2) Gilbert and Fournier's type, where the spleen is more enlarged than the liver; (3) The microsplenic form, where the spleen is little, if at all, enlarged; (4) finally, cases are rarely seen where the liver is atrophied. These varied types show persistent jaundice and are due to varied infections of the bile passages by micro-organisms. The second type occurs most often in children. T. W. Keown [23] recommends sodium glycocholate in diseases of the liver, as bile acids are the only drugs by which the excretion of both the solid and liquid parts of the bile are increased. In the icteroid state of alcoholics, in torpid liver, especially with neurasthenic conditions, in deficient absorption of fats, as in typhoid and pulmonary convalescents, and in the presence of gall stones the drug acts advantageously.

**Congenital Obliteration of Bile Ducts.**—Several instances have been given in former numbers of this journal, and in the *Lancet* of last autumn the deaths of several children in one family were recorded from jaundice, beginning at or soon after birth. W. T. Cocking [24] brings forward a case of jaundice lasting 50 years, which seems to be one of the rare instances where such children have survived. The jaundice began when the patient was three weeks old, and has lasted to the present time. She has been twice married, and one of her children died of jaundice at four months. A combination of diabetes and jaundice is reported by Teleky,[25] which may be compared with the *diabète bronzé* of the French writers. In Teleky's cases a diabetes arose from disease of the pancreas, and the latter led also to obstruction of the ductus communis and severe jaundice. When this reached a high degree the glycosuria disappeared, but the cases ended fatally. Hay's test for the presence of bile acids is carried out by dropping a little of the flowers of sulphur on the suspected urine. If bile is present the sulphur sinks. Spivak [26] finds that to give accurate results the urine must have no bubbles or cloudiness and be quite cold. He obtained the reaction also in advanced tuberculosis and in pregnancy, but it does not appear that he confirmed the test in these cases by any other. Hamel [27] obtains an early indication of jaundice by catching 15–20 drops of blood from the patient in a tube and allowing it to clot. The serum shows a tinge of yellow if the faintest trace of bile is present.

**Weil's Disease.**—Eckardt [28] reports two cases in which a very strong Widal reaction was observed. The blood of jaundiced persons has always some agglutinative effect, and the author asks whether it might not act as a protection in the case of typhoid. If so, it is possible that Weil's disease is simply typhoid which has aborted owing to the development of jaundice. Epidemics of it have been noticed where typhoid was present.

**Cirrhosis.**—G. C. Sears and F. T. Lord [29] give an important analysis of 78 cases of cirrhosis in the Massachusetts Hospital which shows that the liver affection is only one part of a systemic poisoning. The enlargement of veins and of the spleen are partly due to obstruction, but also, in a large degree, to the general poisoning, for they may occur with slight affection of the liver. So, too, the ascites, which depends partly on the peritonitis present. Indeed, hæmorrhagic effusions into the pleuræ and peritoneum have been produced by alcoholism. Large and small livers were found independently of the amount of cirrhosis, and the changes in other organs were as frequent in the early as in the advanced cases. Alcohol was the cause in 67 cases, syphilis in 4, and malaria in 3. In 49 arteriosclerosis appeared as well as cirrhosis, and in 23 of the 78 chronic nephritis. Parkes Weber [30] compares cirrhosis arising from obstruction of the gall ducts to interstitial nephritis due to obstruction of the ureters. In either case, though the obstruction is incomplete, there may be fibrosis of the organ above. He gave cases where multilobular cirrhosis arose thus, and acknowledged that various forms, such as those described above by Lereboulet, occurred without gall-stone obstruction. Rolleston argues that septic obstruction from gall-stones undoubtedly causes cirrhosis, but aseptic obstruction can only act like the causes of hydronephrosis in the kidney. Hale White [31] points out that alcohol *per se* does not cause cirrhosis, thus it fails to produce the disease in animals. The disease is rare in Scotland and common in London, and it occurs in children where alcohol can be excluded. Generally it is due to alcoholic drinks which contain something besides alcohol. However, we must not allow that true cirrhosis is due to syphilis, malaria, or heart failure, or to obstruction. In true cirrhosis due to alcoholic drinks the liver may be large or small according to the stage of the disease, and in more than a third of the cases there are no symptoms of the disease at all. Ascites is not due merely to pressure on the portal vein only, but to some toxin. So too the jaundice and swelling of the feet cannot be due to mere pressure. Other effects of the toxin are anæmia, hæmorrhages, pleural effusions, and coma, all of which may appear in cirrhosis. In uncomplicated cirrhosis if ascites appears the patient is near his end. When patients survive one or two tappings the disease is not simple cirrhosis, but a chronic peritonitis is present. Still this view is opposed by many authorities. He adds that a general perihepatitis is usually part of a chronic peritonitis. In considering the question of the existence of primary malignant disease of the liver, he finds from the Guy's records that it occurs once to 17 or 20 cases of secondary infection, so that it is not quite so rare as supposed.

**Acholia.**—Cheadle[32] discusses a group of cases seen chiefly in teething children, but also in adults who suffer from "Sprue" or tropical white diarrhœa. Gee described the affection as cœliac disease. There is no jaundice but the stools are pure white or clay-coloured, stinking, fatty, and very copious. The condition may last for months or years, the patient loses weight and the abdomen is distended. Temporary acholia may occur in gouty persons and those who live high or have Bright's disease, but the long continued cases are rare. It seems that the absence of colour is not due to pancreatic disease, for colour is restored by giving ox-gall by the mouth. There is, Cheadle thinks, a paralysis of the liver secretion, produced by chill or a nerve reflex from teething. Pancreatised foods, antiseptics, iron and arsenic with the liquor hydrarg. seem to aid recovery.

[22] Les Cirrhoses Biliares. [23] Merck's Archives, Sept. [24] Quart. Med. Jour., Feb. [25] Ibid., p. 161. [26] Med. Rec., Sept. 20. [27] Brit. Med. Jour., Dec. 27. [28] Amer. Jour. Med. Sci., Jan. [29] Lancet, Oct. 25. [30] Brit. Med. Jour., Dec. 6. [31] Brit. Med. Jour., March 7. [32] Lancet, May 30.

